# Economic evaluation of culprit lesion only PCI vs. immediate multivessel PCI in acute myocardial infarction complicated by cardiogenic shock: the CULPRIT-SHOCK trial

**DOI:** 10.1007/s10198-020-01235-3

**Published:** 2020-10-07

**Authors:** Jose Antonio Robles-Zurita, Andrew Briggs, Dikshyanta Rana, Zahidul Quayyum, Keith G. Oldroyd, Uwe Zeymer, Steffen Desch, Suzanne de Waha-Thiele, Holger Thiele

**Affiliations:** 1 Health Economics and Health Technology Assessment, Institute of Health and Wellbeing, University of Glasgow, 1 Lilybank Gardens, Glasgow, G12 8RZ UK; 2 London School of Hygiene & Tropical Medicine, London, UK; 3 BRAC James P Grant School of Public Health, BRAC University, Dhaka, Bangladesh; 4 West of Scotland Regional Heart and Lung Centre, Golden Jubilee National Hospital, Glasgow, UK; 5 Klinikum Ludwigshafen and Institut für Herzinfarktforschung, Ludwigshafen, Germany; 6 Heart Center Leipzig, University of Leipzig and Leipzig Heart Institute, Leipzig, Germany; 7 University Heart Center Lübeck, University Hospital Schleswig-Holstein (UKSH), Lübeck, Germany

**Keywords:** Culprit-shock trial, Economic evaluation, Pre-trial model, Decision analytic modelling, I10

## Abstract

**Background:**

The CULPRIT-SHOCK trial compared two treatment strategies for patients with acute myocardial infarction and multivessel coronary artery disease complicated by cardiogenic shock: (a) culprit vessel only percutaneous coronary intervention (CO-PCI), with additional staged revascularisation if indicated, and (b) immediate multivessel PCI (MV-PCI).

**Methods:**

A German societal and national health service perspective was considered for three different analyses. The cost utility analysis (CUA) estimated costs and quality adjusted life years (QALYs) based on a pre-trial decision analytic model taking a lifelong time horizon. In addition, a within trial CUA estimated QALYs and costs for 1 year. Finally, the cost effectiveness analysis (CEA) used the composite primary outcome, mortality and renal failure at 30-day follow-up, and the within trial costs. Econometric and survival analysis on the trial data was used for the estimation of the model parameters. Subgroup analysis was performed following an economic protocol.

**Results:**

The lifelong CUA showed an incremental cost effectiveness ratio (ICER), CO-PCI vs. MV-PCI, of €7010 per QALY and a probability of CO-PCI being the most cost-effective strategy > 64% at a €30,000 threshold. The ICER for the within trial CUA was €14,600 and the incremental cost per case of death/renal failure avoided at 30-day follow-up was €9010. Cost-effectiveness improved with patient age and for those without diabetes.

**Conclusions:**

The estimates of cost-effectiveness for CO-PCI vs. MV-PCI have been shown to change depending on the time horizon and type of economic evaluation performed. The results favoured a long-term horizon analysis for avoiding underestimation of QALY gains from the CO-PCI arm.

**Electronic supplementary material:**

The online version of this article (10.1007/s10198-020-01235-3) contains supplementary material, which is available to authorized users.

## Introduction

Most patients with acute myocardial infarction (MI) complicated by cardiogenic shock have multivessel disease with an estimated annual incidence of cardiogenic shock of > 45,000 patients in Europe and > 30,000 in the United States [1]. Mortality rates for patients with multivessel coronary artery disease are higher than for patients with single vessel disease ranging between 40 and 70% [2–4]. Two alternative strategies for the treatment of multivessel disease could be considered. On the one hand, early mechanical reperfusion of the culprit lesion by percutaneous coronary intervention (PCI) plus staged revascularisation of all other remaining significant lesions could be performed. On the other hand, a strategy of immediate PCI of all significant stenoses could be followed. Previous European guidelines for the management of acute ST-segment elevation MI recommended immediate multivessel PCI based mainly on pathophysiological considerations and observational data as there was no data from randomised clinical trials [5]. Previous guidelines from the United States considered revascularization of both culprit and non-culprit arteries during the same procedure to be highly appropriate [6].

The multicentre, randomised CULPRIT-SHOCK trial investigated whether culprit vessel only PCI (CO-PCI) with additional staged revascularisation if indicated was more effective than immediate multivessel PCI (MV-PCI) for patients with cardiogenic shock complicating acute myocardial infarction. At 30 days, the composite primary endpoint of death and severe renal failure with renal replacement therapy was significantly lower in the CO-PCI group (45.9%) than in the MV-PCI group (55.4%) [Relative risk (RR) of 0.83; 95% confidence interval (CI) 0.71–0.96] [7]. One year after randomisation the same figures were 52.0% and 59.5% (RR 0.87; 95% CI 0.76–0.99), respectively [8]. These results provoked a reassessment of the optimal revascularisation strategy in patients with STEMI, multi-vessel disease and cardiogenic shock [9, 10]. European revascularization guidelines now recommend “culprit lesion-only PCI as the default strategy in these patients [11].

This study contributes to the discussion by presenting the economic evaluation of the alternative revascularization strategies within the CULPRIT-SHOCK trial. A cost effectiveness analysis (CEA) and cost utility analysis (CUA) is performed to estimate the consequences on medical outcomes, quality adjusted life years (QALYs) and costs during the trial period. A pre-trial decision analytic model was used to analyse long term cost-effectiveness of CO-PCI vs. immediate MV-PCI [12]. The need for an economic analysis is justified given different consequences for healthcare costs. For example, there was more repeat revascularisation of non-culprit lesions and rehospitalisation for heart failure in the CO-PCI arm than in the MV-PCI group but more patients died or required renal replacement therapy in the MV-PCI group [8].

## Methods

### Population, setting and location, and comparators.

The CULPRIT-SHOCK trial population are patients with acute MI, cardiogenic shock, and multivessel coronary artery disease from 83 European centres. Seven hundred and six patients were randomised to the two different revascularization strategies: CO-PCI or immediate MV-PCI. Full informed consent was obtained for 686 patients. The details of the methods and design of the trial have been published previously as well as the clinical outcome for the 30-day primary endpoint and the 1-year follow-up [7, 8, 13].

### Economic evaluation and time horizon

The cost utility analysis (CUA) estimated all costs and quality adjusted life years (QALYs) based on a pre-trial decision analytic model taking a lifelong time horizon. In addition, a within trial period CUA estimated QALYs and cost by running the model for up to 1 year after randomisation. The cost effectiveness analysis (CEA) used the composite primary outcome, mortality and renal failure at 30-day follow-up, and the within trial costs.

### Perspective, guidelines and discounting

Given that a majority of patients were from Germany, the economic evaluation took a German societal and National Health Service perspective, considering productivity costs in addition to health care costs. The economic analysis followed guidelines of the Institute for Quality and Efficiency in Health Care, good practice in health technology assessment and the CULPRIT-SHOCK economic protocol [12, 14, 15]. The analysis was based on an intention-to-treat principle. A 3% discount rate was applied to future costs and QALYs.

### Measurement and valuation of costs and health outcomes

Resource use was collected at baseline, 30-day, 6-month and 12-month follow-up. The resources used were valued at unit costs provided by the Institute for the Hospital Remuneration System (InEK) using diagnosis related groups (DRGs). Medication prices were obtained from the German Institute of Medical Documentation and Information (DIMDI).^1^ Unit costs from the literature were used where required [16–22] (details in supplementary material).

Survival data from the trial was used to estimate the probability of different health conditions. Time spent in each health condition was weighted by quality of life using the EuroQol five-dimension three-level (EQ5D) instrument, reported by patients at 30-day, 6-month and 12-month follow-up [23]. The EQ5D was valued by the German value set [24].

### The model

A published pre-trial cohort model allowed us to estimate costs and effects for the economic evaluation [12].

#### Structure: decision tree and Markov model

**Fig. 1:**
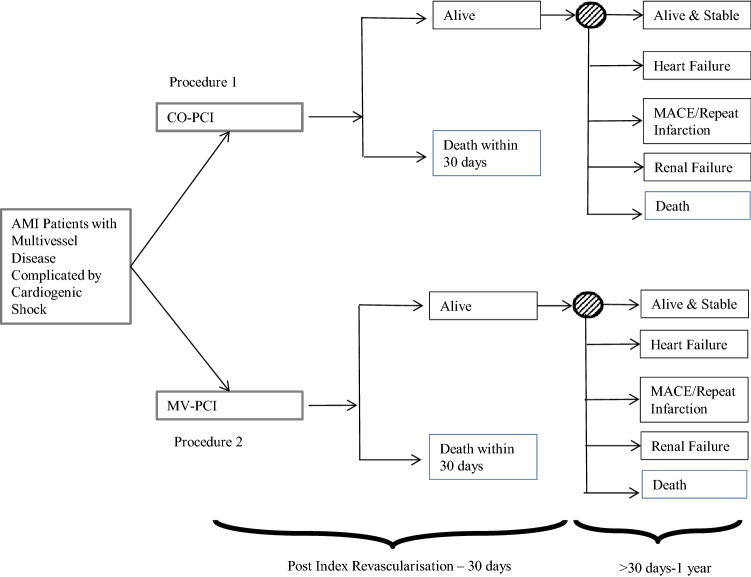
Decision tree. Previously published in Quayyum et al. [12]

**Fig. 2:**
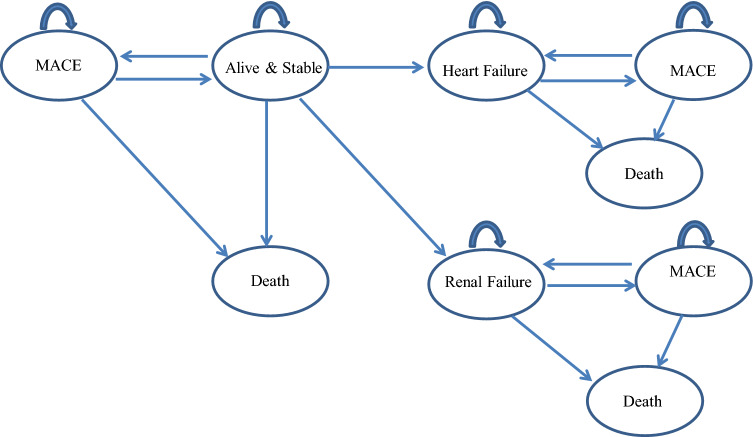
Markov model. Previously published in Quayyum et al. [12]

The model is shown in Fig. 1, for the 1-year decision tree, and Fig. 2, for long-term Markov model. For each revascularization strategy, the first node in Fig. 1 represents the uncertainty for a patient to die or survive 30 days after the medical intervention. All those who survive the first month go through a second node that gives place to different endpoints: death, renal failure, heart failure, major adverse cardiac event (MACE) and alive and stable (A&S). The health states are assumed mutually exclusive, i.e., a patient could only experience one health outcome. Given that a patient could experience more than one health state in practice, we applied the next definitions. A patient was in Death state if they died from any cause. Renal failure was the state for a patient receiving renal replacement therapy and surviving 1 year after randomisation. A patient was in the heart failure state if re-hospitalised for that reason and was not dead or in renal failure. MACE included all those patients who suffered a major adverse cardiovascular event (i.e., MI, stroke or revascularisation) during the first year after the treatment but did not suffer heart failure, renal failure or death. Finally, A&S was the residual health condition for all patients that are not included in any of the remaining states.

Patients who survive 1 year after randomisation move to the Markov model stage shown in Fig. 2. A monthly-cycle model allows patients to move to different health states in the long-term. Patients can die or remain in their chronic condition: renal failure, heart failure and A&S. Patients with MACE at the end of the decision tree period are assumed to move back to health state A&S if MACE does not recur and they do not die. All patients in chronic states can suffer from MACE and change condition in the next cycle. Only patients who remain A&S can transit to renal or heart failure, but the opposite pathway is not possible. Finally, patients can switch to a death state from any health state. All the transitions are assumed to happen at the beginning of each period.

#### Parameters

The parameters of the model can be classified as:

- Transition probabilities
- Utilities
- Cost parameters

##### Transition probabilities

Transition probabilities for the decision tree were estimated from raw frequencies of each health state at 30-day and 12-month follow-up. The transition probabilities for CO-PCI were computed by applying relative risks of death, renal failure, heart failure, and MACE to transition probabilities for MV-PCI. These relative risks were estimated for each event separately using trial data.

Transition probabilities in the long term are probability of death from any health state; renal failure; MACE, and rehospitalisation for heart failure. Survival analysis was applied to the within trial data to estimate long-term probabilities for the Markov model. Parametric models were used to fit the within trial data following methodological guidelines in the literature [25]. Transition probabilities are estimated conditional on patient characteristics (age, gender and whether the patient is diabetic) and trial arm. Mortality estimated for the Markov model is also conditional on health states. To avoid unrealistic predictions of death probabilities in the long term, survival analysis was used to predict mortality for the first cycle of the Markov model, thereafter, probability of death was assumed to change proportionally to mortality rates given by life tables [26].

##### Utilities

The model incorporated quality of life by weighting time on each health state by a health utility score estimated from trial data. The EQ5D health utilities for each patient were used to estimate a random effect econometric model including the health state as explanatory variable; the individual patient was included as the panel variable. This model controlled for other variables such as: age, gender, diabetes disease, body mass index (BMI), history of cardiovascular disease and risk factors. Utilities could vary among health states but not between trial arms. Differences in quality of life between arms came from differences in frequency of health states. Finally, a zero utility was assigned to the death-state.

The health utility attached to MACE in the long term was changed with respect to the utility of MACE in the decision tree. A substantial proportion of MACE in the first year were patients having staged revascularization due to a medical decision, taken within the scope of the revascularization strategy. It was considered that the health utilities derived for those patients would not be an accurate prediction for patients suffering a cardiac event in the future. For the long term, the same utility model explained above was estimated, where only patients with urgent revascularization, MI or stroke were included in the MACE state.

##### Costs

The estimation of costs included in the model varied for the first-year decision tree and the long-term Markov model. For the first year, a within trial cost was estimated for each arm, where all the resources used were valued by the unit costs. The list of costs collected included: repeat revascularization/PCI; hospitalization after PCI, differentiating between intensive care unit (ICU) or normal ward; inpatient hospitalization and emergency room visits at follow-up; medications; stents; angiography; internal cardioverter defibrillators (ICD); extracorporeal membrane oxygenation (ECMO) procedures; intra-aortic balloon pump (IABP) procedures; left ventricular assist devices (LVAD); heart transplants; investigations; renal replacement therapy, and; productivity losses. All the cost information was reported in the electronic case report form (eCRF) completed by the treating clinicians; except for inpatient hospitalization, visits to emergency room and productivity losses that were reported by patients and confirmed by hospital and/or general practitioners reports.

The estimation of some costs is explained in more detail below:

- *Hospitalization costs* Hospitalization after index PCI, ICU and normal ward, was collected using eCRF. In addition, patients reported inpatient hospitalization at 30-day follow-up. To avoid double counting in hospitalization costs, we did not include self-reported inpatient hospitalization at 30-day follow-up for the computation of the cost of each treatment alternatives.
- *Renal replacement therapy* All the patients that received renal replacement therapy were attached a one-time cost for therapy costs. If the patient survived 1 year after randomisation, an additional cost of dialysis was included to account for cost of dialysis for that period.
- *Angiography* A cost for diagnosis related group (DRG) was considered for each patient undertaking angiography. To account for the number of angiographies and time spent, we assigned the DRG cost in proportion to the total time the patient spent in a fluoroscopy.
- *Productivity costs* Patients reported the number of days of work lost. A friction period of 60 working days (8 h) was assumed for patients who left work for more than 60 days or dies before age 65. For patients that left paid work but returned to unpaid work after revascularization, a cost of 30 working days was imputed. If the patient reduced working hours at work, a cost of 10 working days was assumed. Patients that did not work before randomization had zero cost.
- *Medications* We had information about whether a patient was prescribed or administered a specific medication during hospitalization at ICU or normal ward after PCI. We assumed that the medication was administered for the time spent at the hospital. A standard dose per day was assumed following opinion from experts involved in the trial and the British National Formulary (BNF).

In the long-term model, a cost was attached to each cycle conditional on health state based on a random effect econometric model that estimated hospitalization and emergency room costs using information for 6- and 12-month follow-up, where the patient was the panel variable. Explanatory variables and specification of this model are the same as for the utility model explained above.

The unit costs considered in the analysis are shown in the Online Appendix.

### Missing values and outliers

Missing values for hospitalization and emergency room visits were imputed algorithmically. Missing values were converted to zero for all those patients that died before they could have any hospitalization at each follow-up period. In other cases, missing values were replaced by the average value conditional to survival/death at each specific follow-up. The complete case analysis was performed for all other variables used in the study.

Outliers were identified and eliminated when statistically/unrealistically diverged from the sample distribution.^2^

### Cost effectiveness outcome

The economic evaluation was intended at estimating the incremental cost effectiveness ratio (ICER), where the effectiveness measure is either QALY or percentage of death/renal failure for the CUA or CEA, respectively. In addition, the net monetary benefit was estimated for a range of policy relevant monetary values of a QALY to build the cost effectiveness acceptability curves (CEACs).

### Probabilistic sensitivity analysis

Monte Carlo simulation was used to perform the probabilistic sensitivity analysis. The model parameters were assumed to vary according to specific distributions with parameters estimated from trial data. Lognormal distributions were assumed for the relative risks and beta distributions for absolute risk. For costs, Gamma distributions were used. Finally, Beta distributions were considered for utilities and incremental utilities.

### Subgroup analysis

Per protocol predefined subgroup analysis was performed to study the heterogeneity of the results for: age groups (< 50 years, 50–75 years, > 75 years); sex, and patients with/without diabetes. The parameters of the model were adapted to each subgroup. The treatment effect on decision tree probabilities estimated for the total sample was applied to baseline probabilities for each trial subgroup. Markov model transition probabilities were also specific for each subgroup according to the survival analysis estimations. Health utilities were adjusted to the average of each subgroup for patients in the state A&S. The absolute differential effect of each health state was assumed the same for all subgroups. Costs were estimated/computed for each subgroup separately considering the resource use within 1 year after randomisation and the frequency of each health state in long term costs.

### Software

The model results were obtained using the package ‘*heemod’* for the statistical software R [27, 28]. All statistical and econometric analyses were performed using STATA 14.0 (StataCorp, TX, USA) [29].

## Results

### Base-case

#### Transition probabilities

The estimated frequencies for each health state of the decision tree are shown in Table 1. At 30-day follow-up, the death rate was lower in the CO-PCI arm than in the MV-PCI group (43.3% v. 51.5%) with a statistically significant relative risk (0.84, *p* value = 0.033). Conditional to surviving the first 30 days after randomisation, death rate was similar in both groups between 30 days and 12-month follow-up (11.8% and 10.8%, respectively; relative risk of 1.09, *p* value = 0.777). The conditional probability of being in MACE is significantly higher for the culprit-only revascularization strategy than for the multivessel PCI group (44.6% vs. 19.9%). A closer inspection reveals that these differences are driven by a higher rate of repeat revascularization performed in the CO-PCI arm; the rate of stroke or MI was not significantly different between groups as reported in the main efficacy analysis [8]. Finally, the CO-PCI strategy had a higher probability of heart failure (relative risk of 2.55, *p* value = 0.099) and a lower frequency of renal failure (relative risk of 0.66, *p* value = 0.403), conditional to being alive 30 days after randomisation. Therefore, the number of people being in A&S, conditional to surviving the first 30 days after revascularization, is statistical significantly higher for the MV-PCI arm.

**Table 1:** Probability of endpoints of decision tree

	CO-PCI %	MV-PCI %	Relative risk	*p* value
Post index revascularization: 30 days				
Death	43.3	51.5	0.84	0.033
30 days to 1 year				
Death	11.8	10.8	1.09	0.777
Renal failure	3.6	5.4	0.66	0.403
Heart failure	6.2	2.4	2.55	0.099
MACE	44.6	19.9	2.24	< 0.001
A&S	33.8	61.4	0.55	< 0.001
*N*	344	342		

Probabilities for the period 30 days to 1 year after randomization are conditional to survival 30 days after randomization, i.e., number of patients in each health state divided by the number of patients alive at 30-day follow-up

Details of survival analysis for the estimation of transition probabilities in the long term are described in the Online Appendix. Different parametric survival models were considered: exponential, Gompertz, Weibull, loglogistic and lognormal. Given the patterns shown for mortality rates, with deaths concentrated in the first 30 days after randomisation, death transition probabilities were estimated by fitting data for the period 30 days to 1 year after randomisation. The Weibull model was the best fit according to the Akaike’s information criterion (AIC) and Bayesian information criterion (BIC). No statistically significant differences between trial arms were estimated (*p* value = 0.953). Only a statistically significant higher mortality hazard was estimated for patients with diabetes with respect to the non-diabetes subgroup (*p* value = 0.01). Hazard rate also increased (no statistical significance, *p* value > 0.05) for patients with health conditions (renal failure, heart failure and MACE), older age and male gender. The exponential model was the best fit for the estimation of heart failure hazard. Only a significantly higher hazard was estimated for the CO-PCI arm vs. the MV-PCI group (*p* value = 0.01). In the case of MACE, the best-fit model was lognormal and a higher hazard for the CO-PCI strategy was estimated (*p* value = 0.048). Finally, no new patients required renal replacement therapy after the first 19 days from randomisation, so the risk of renal failure was assumed zero in the long term.

##### Health utilities

Table 2 shows the EQ5D health utilities by arm and follow-up. There were no statistically significant differences between arms at any follow-up. Nonetheless, quality of life for both trial arms improve over time as health utilities at 12-month follow-up tend to be higher than at 6-month and at 30-day follow-up. Finally, Table 3 reports the results for the utility model estimating differences between health states. Our estimates suggest that there was not any statistically significant effect of any health condition (heart failure, renal failure, MACE) with respect to A&S. Nonetheless, health utilities estimates were higher, for MACE, and lower, for renal failure and heart failure, with respect to A&S. The effect of suffering MACE on health utilities was not statistically significant when only patients with urgent revascularization, MI or stroke were included (*p* value = 0.707). For the subgroup variables, we estimated a lower health utility for older patients, females and diabetes condition (no statistically significant at 10%). The only statistically significant control variable was history of CVD with a negative impact on utility (*p* value = 0.052).

**Table 2:** EQ5D health utilities

Follow-up	CO-PCI		MV-PCI		Incremental	
	Mean	*N*	Mean	*N*	Mean	*p* value
30 days	0.756	171	0.728	143	0.028	0.430
6 months	0.833	161	0.815	138	0.018	0.533
12 months	0.842	155	0.845	132	− 0.004	0.891

The number of observations for each group are those patients who survive to each follow-up period with no missing information

**Table 3:** Health utility model

	Coef	*p* value
Health state (ref. A&S):		
HF	− 0.051	0.294
MACE	0.036	0.130
RF	− 0.085	0.161
Control variables		
Age (ref. < 50)		
50–75	− 0.0003	0.996
> 75	− 0.079	0.208
Male	0.041	0.242
Diabetes	− 0.036	0.264
BMI (ref. normal or underweight)		
Overweight	− 0.013	0.663
Obese	− 0.0004	0.991
CVD history	− 0.057	0.052
Risk factor	0.041	0.308
Cons	0.773	0.000
*N*—number of patients	322	
*N*—number of patient-follow-ups	875	

The constant of the model represents a female patient, in the A&S state, younger than 50, with no diabetes, normal or underweight, no CVD history and no risk factors. The coefficient of MACE changed to 0.0104 (*p* value = 0.707) if only patients with urgent revascularization, MI or stroke were included

##### Costs

Resource use and monetary costs for the first year after randomisation are shown in Table 4. The cost was not statistically significantly different between arms for most categories. Nonetheless, some patterns were consistent with the clinical results and with the characteristics of each revascularization strategy. CO-PCI patients incurred more in repeat revascularization and ICU costs, while patients in MV-PCI had more costs related to renal replacement therapy (continuous dialysis), number of stents and angiography. Total cost did not significantly differ between revascularization strategies (*p* value = 0.679), although it was higher for the CO-PCI arm on average by €841 per patient.

**Table 4:** Average resource use and costs during 1 year after randomization

	Resource use (units/patient)			Cost (€/patient)			
	CO-PCI	MV-PCI	Incremental	CO-PCI	MV-PCI	Incremental	*p* value
Number of urgent/staged PCIs	0.324	0.093	0.231	1052	302	751	< 0.001
Hospitalizations							
ICU nights	7.832	7.526	0.305	7522	7229	293	0.679
Normal ward nights	5.246	5.820	− 0.574	1500	1664	− 164	0.432
Impatient days	2.138	3.758	− 1.620	611	1074	− 463	0.243
Emergency room visits	0.381	0.259	0.122	149	101	48	0.486
Number of renal rep. therapy							
Continuous dialysis	0.044	0.093	− 0.049	35	75	− 40	0.013
Hemodiafiltration	0.031	0.028	0.003	29	26	3	0.806
Hemofiltration	0.025	0.034	− 0.009	24	33	− 9	0.494
Intermittent dialysis	0.016	0.012	0.003	4	3	1	0.731
Number renal failure long term	0.019	0.028	− 0.009	1211	1806	− 594	0.441
Stents	2.688	3.582	− 0.894	185	246	− 61	< 0.001
Angiography (minutes in fluoroscopy)	18.353	22.540	− 4.187	426	523	− 97	< 0.001
Number of ICDs	0.040	0.043	− 0.003	113	121	− 8	0.857
Number of ECMOs	0.062	0.102	− 0.040	600	984	− 384	0.066
Number of IABPs	0.090	0.080	0.010	115	102	12	0.655
Number of LVADs	0.140	0.118	0.023	6046	5074	972	0.407
Number of heart transplants	0.003	0.000	0.003	62	0	62	0.316
Number of investigations procedures							
Scintigraphy	0.003	0.000	0.003	0	0	0	0.316
Stress-echocardiogram	0.000	0.015	− 0.015	0	0	0	0.025
MRI	0.006	0.000	0.006	2	0	2	0.156
Days of medications							
Aspirin	12.545	13.250	− 0.706	0	0	− 0	0.570
Clopidogrel	6.274	6.666	− 0.392	70	74	− 4	0.720
Prasugrel	3.625	4.114	− 0.490	5	6	− 1	0.531
Ticagrelor	4.777	4.455	0.322	7	6	0	0.692
Vitamin-K-antagonists	1.628	1.407	0.221	3	3	0	0.687
GP IIb/IIIa-inhibitors	0.224	0.220	0.004	42	41	1	0.891
UF heparin	10.439	10.524	− 0.085	0	0	− 0	0.942
LMW heparin	1.620	2.584	− 0.964	25	39	− 15	0.118
Bivalirudin	0.050	0.068	− 0.018	25	34	− 9	0.326
Fondaparinux	0.380	0.573	− 0.193	0	1	− 0	0.566
Beta-blocker	10.465	10.035	0.430	94	90	4	0.713
ACE-inhibitor/AT-II-antagonist	9.503	8.743	0.760	14	13	1	0.499
Statins	11.095	11.043	0.052	43	43	0	0.965
Calcium-antagonist	1.734	1.308	0.426	0	0	0	0.416
Aldosterone-antagonist	3.690	4.082	− 0.392	4	5	− 0	0.664
Diuretics	9.042	8.048	0.994	0	0	0	0.376
Edoxaban	0.022	0.000	0.022	0	0	0	0.316
Apixaban	0.257	0.310	− 0.053	1	1	− 0	0.811
Rivaroxaban	0.595	0.121	0.474	1	0	1	0.035
Dabigatran	0.204	0.337	− 0.133	0	0	− 0	0.596
Catecholamine therapy	6.406	6.657	− 0.251	6	7	− 0	0.133
Work hours lost	154.642	139.022	15.620	5343	4803	540	0.365
Total				25,371	24,531	841	0.679
*N*	321	323		321	323		

Medication are expressed in days receiving the standard dose, except for the case of GP IIb/IIIa-inhibitors, UF heparin, bivalirudin and catecholamine therapy, where proportion of patient receiving medication is shown

The cost model is shown in Table 5, where hospitalization and emergency room costs reported at 6-month and 12-month follow-up were regressed against health states and control variables. A higher cost is estimated for all the health conditions compared to A&S, but only estimates for heart failure are statistically significant at 10%. In the long-term Markov model, a monthly cost was attached to each cycle, conditional on health state, based on the estimates.

**Table 5:** Cost model

	Coef	*p* value
Health state (ref. A&S)		
HF	1922.7	0.095
MACE	92.6	0.884
RF	395.6	0.723
Control variables		
Age (ref. < 50)		
50–75	254.8	0.800
> 75	224.4	0.843
Male	509.0	0.425
Diabetes	6.2	0.992
BMI (ref. normal or underweight)		
Overweight	27.0	0.962
Obese	− 447.3	0.531
CVD history	425.9	0.430
Risk factor	260.6	0.718
Cons	− 19.1	0.988
*N*—number of patients	322	
*N*—number of patient-follow-ups	634	

The constant of the model represents a female patient, in the A&S state, younger than 50, with no diabetes, normal or underweight, no CVD history and no risk factors. MACE included patients with urgent revascularization, MI or stroke

#### Cost-effectiveness results

The results for the base-case analysis are shown in Table 6 rounded to 3 significant figures. The percentage of death/renal failure was significantly higher in the MV-PCI arm (0.0933; 95% CI 0.0225, 0.159). As a result, the average ICER for the CEA was €9010 per case of death/renal failure avoided. With respect to the CUAs, the results are based on the parameters estimated and summarised for the decision tree and Markov model in the Online Appendix. If we restrict the analysis to the within trial period, the CO-PCI strategy entails an increase of 0.0577 QALYs per patient (95% CI − 0.00275, 0.114) with an average ICER about 14,600 €/QALY. However, a huge uncertainty remains as shown in the confidence interval.

**Table 6:** Economic evaluation results: costs, effects and ICERs

Intervention strategies	Costs (€/patient)		Effect (per patient)		ICER	
	Mean	[95% CI]	Mean	[95% CI]	Mean	[95% CI]
CEA			% death/renal problem (30-day FU)		€/case	
CO-PCI	25,400	[22,600, 28,200]	0.541	[0.457, 0.615]		
MV-PCI	24,500	[21,800, 27,500]	0.447	[0.393, 0.5]		
CO-PCI vs. MV-PCI	841	[− 3360, 4780]	0.0933	[0.0225, 0.159]	9010	[− 45,400, 83,200]
CUA (1-year FU)			QALYs		€/QALY	
CO-PCI	25,400	[22,600, 28,200]	0.398	[0.319, 0.478]		
MV-PCI	24,500	[21,800, 27,500]	0.34	[0.285, 0.394]		
CO-PCI vs. MV-PCI	841	[− 3360, 4780]	0.0577	[− 0.00275, 0.114]	14,600	[− 111,000, 178,000]
CUA (lifelong)			QALYs		€/QALY	
CO-PCI	27,200	[23,500, 37,700]	2.94	[1.09, 5.49]		
MV-PCI	25,100	[22,400, 29,,400]	2.64	[0.951, 4.83]		
CO-PCI vs. MV-PCI	2060	[− 2370, 10,500]	0.293	[− 0.69, 1.51]	7010	[− 92,200, 107,000]

Effectiveness measure for CEA is % reduction of composite outcome death or renal-replacement therapy at 30-day follow-up. Figures are rounded to 3 significant figures

*CO-PCI* culprit only percutaneous coronary intervention, *MV-PCI* multivessel percutaneous coronary intervention.

**Fig. 3:**
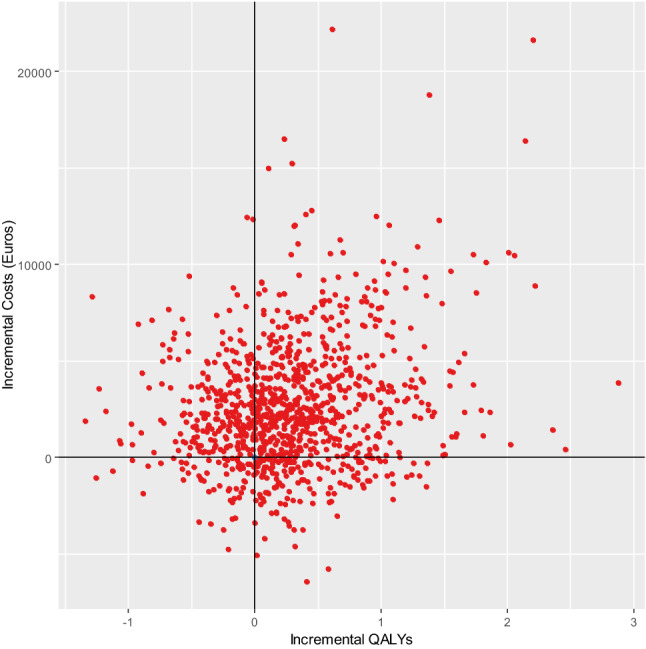
Cost Effectiveness Plane. CV-PCI vs. MV-PCI. Lifelong CUA base case analysis

**Fig. 4:**
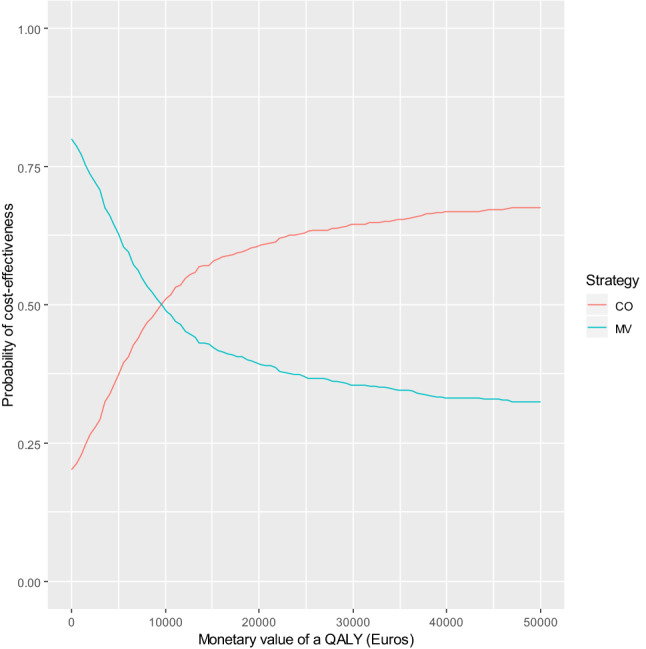
CEAC. Lifelong CUA base case analysis

The lifelong analysis shows non-significant differences in costs and QALYs. The average cost for CO-PCI arm are about €2060 (95% CI − 2370, 10,500) higher than for the MV-PCI arm, which is more than double the difference estimated for the within trial period. Nonetheless, the lifelong QALY increment for the CO-PCI strategy is 0.293 (95% CI − 0.69, 1.51) per patient, which is about five times higher than the figure estimated for the first year after randomisation. The consequence of considering a long-term perspective is an average ICER approximately 7010 €/QALY, less than half the estimate from the within trial CUA. The uncertainty around the estimates is represented in the cost effectiveness plane (CEP) and cost acceptability curves (CEAC) shown in Figs. 3 and 4, respectively. The simulation points are spread over the four quadrants of the CEP. However, most of the simulation points (about 58%) are in the upper-right quadrant of the CEP implying that CO-PCI tended to be both more costly and effective. Finally, the CEAC shows that the CO-PCI is the most (less) likely cost-effective strategy for any monetary value of a QALY higher (lower) than €9000. Indeed, the probability of CO-PCI being cost-effective is higher than 64% for any threshold above 30,000 €/QALY.

### Subgroup analysis

The subgroup analyses for age, gender and diabetes can be compared in Table 7. The results for the total sample are mainly replicated for all the subgroups, i.e., the CO-PCI strategy is the costliest and most effective. However, some differences between subgroups appear with respect to the cost-effectiveness of the strategies being evaluated. For example, the cost effectiveness of CO-PCI improves for older subgroups. For patients aged > 75 this treatment alternative is dominant, meaning that on average is both more effective and less costly. On the contrary, for the youngest subgroup, age < 50, the average ICER was about 117,000 €/QALY and the probability of CO-PCI being the most cost-effective alternative is lower than 43% for any monetary value of a QALY below €50,000 (see Online Appendix for CEPs and CEACs for subgroups). The cost effectiveness of the CO-PCI decreases for patients with diabetes with an ICER of 14,700 €/QALY compared to patients without this condition with an ICER of 5680 €/QALY. No substantial differences in cost effectiveness have been found between gender groups.

**Table 7:** CUA (lifelong). Subgroups

Intervention strategies	*N*	Costs (€/patient)		Effect (per patient)		ICER	
		Mean	[95% CI]	Mean	[95% CI]	Mean	[95% CI]
Age < 50				QALYs		€/QALY	
CO-PCI	17	51,900	[38,600, 74,700]	7.88	[2.18, 13.5]		
MV-PCI	16	42,800	[31,100, 55,500]	7.8	[2.23, 13.5]		
CO-PCI vs. MV-PCI	33	9090	[− 8240, 30,400]	0.0774	[− 2.16, 1.97]	117,000	[− 286,000, 153,000]
Age 50–75				QALYs		€/QALY	
CO-PCI	212	38,600	[33,400, 52,600]	3.91	[1.45, 7.03]		
MV-PCI	227	36,100	[32,500, 42,300]	3.62	[1.32, 6.21]		
CO-PCI vs. MV-PCI	439	2500	[− 3610, 12,800]	0.295	[− 0.953, 1.73]	8500	[− 84,000, 108,000]
Age > 75				QALYs		€/QALY	
CO-PCI	115	17,500	[13,600, 23,700]	0.947	[0.258, 2.65]		
MV-PCI	99	17,800	[13,500, 23,200]	0.744	[0.275, 1.98]		
CO-PCI vs. MV-PCI	214	− 226	[− 6970, 7010]	0.203	[− 0.335, 1.05]	CO-PCI dominates	
Female				QALYs		€/QALY	
CO-PCI	86	21,800	[15,500, 35,100]	3.21	[1.21, 5.88]		
MV-PCI	75	19,000	[14,700, 25,900]	2.86	[1.04, 5.06]		
CO-PCI vs. MV-PCI	161	2810	[− 4610, 14,200]	0.347	[− 0.734, 1.6]	8080	[− 95,300, 105,000]
Male				QALYs		€/QALY	
CO-PCI	257	29,100	[25,000, 39,500]	2.86	[1.05, 5.65]		
MV-PCI	267	26,900	[23,700, 31,900]	2.58	[0.928, 4.89]		
CO-PCI vs. MV-PCI	524	2160	[− 2880, 10,100]	0.28	[− 0.721, 1.56]	7740	[− 130,000, 94,000]
No diabetes				QALYs		€/QALY	
CO-PCI	241	27,300	[22,700, 39,800]	3.58	[1.39, 6.52]		
MV-PCI	226	25,300	[22,000, 30,700]	3.24	[1.19, 5.72]		
CO-PCI vs. MV-PCI	467	1970	[− 3380, 11,400]	0.346	[− 0.805, 1.69]	5680	[− 82,500, 66,900]
Diabetes				QALYs		€/QALY	
CO-PCI	102	27,200	[21,900, 36,500]	1.88	[0.642, 4.12]		
MV-PCI	116	24,700	[20,300, 30,500]	1.71	[0.615, 3.59]		
CO-PCI vs. MV-PCI	218	2520	[− 5000, 11,400]	0.172	[− 0.605, 1.18]	14,700	[− 169,000, 196,000]

Figures are rounded to 3 significant figures. *N* represents the distribution of participating patients according to subgroup characteristics

*CO-PCI* culprit only percutaneous revascularisation, *MV-PCI* multivessel percutaneous revascularisation.

## Discussion

The within trial data provided evidence for costs, health states distribution and health utilities of CULPRIT-SHOCK patients up to 1 year from randomisation. In addition, econometric and survival analysis estimated parameters for the pre-trial model making the projection of cost effectiveness of CO-PCI vs. MV-PCI in the long term possible. The results showed that it is important to undertake a long-term approach as it derives from the differences between the within trial and lifelong analysis. Only considering within trial analysis, CO-PCI would be cost effective for a monetary value of at least €14,600 per QALY. The long-term analysis reduced the ICER to about €7000 per QALY, indicating an underestimation of QALY gains when a short-term horizon was considered. Given the survival gains estimated in the short term (at 30-day follow-up), cost-effectiveness improves as time horizon increases. Even in the case that mortality rates did not differ between arms in the long term (i.e., after the 1-year trial period), as it seems to be the case here, QALYs would accumulate over time due to short term reduced mortality. This pattern can be found in previous studies [23–32]. Nonetheless, higher incremental costs were estimated for the CO-PCI arm when a lifelong CUA was compared to a within trial analysis; consistently with a higher risk of heart failure and MACE. Finally, subgroup analysis seems to reveal some patterns that are worthwhile to have into account. The cost-effectiveness of CO-PCI vs. MV-PCI increases with age and for the non-diabetes patients. Although results are similar between patients classified by gender.

This study presents the first economic evaluation based on randomised data comparing MV-PCI vs. CO-PCI for patients with acute MI and cardiogenic shock. Cohort or registry studies have limitations for interpreting differences between arms as causal effects. In the context of MI complicated by cardiogenic shock, physician discretion to determine treatment strategy (complete vs. culprit only PCI) has been acknowledged to incorporate a selection bias in the estimations of outcomes [33]. A strength of this analysis is the use of data from the Culprit-Shock trial, avoiding selection bias by randomly allocating patients to treatment groups [9].

The analysis followed a health economic analysis plan (HEAP) that established different details of the economic evaluation like time horizon, outcomes and variables, statistical methods, sources of information and a decision analytic model [12]. The advantages of using HEAPs to guide economic evaluations have been acknowledged in the health economics literature, particularly regarding transparency and biases reduction [34, 35]. Publications of health economic protocols has been increasing in the last few years [36–39]. However, the use of a published pre-trial model introduces an innovative aspect, since few HEAPs contain a detailed decision analytic model for a long-term analysis [40].

The long-term results must be interpreted in line with the strategy used for the estimation of model parameters. For example, statistical measures and visual inspection have been applied to choose parametric survival models that best fitted within trial data. Nonetheless, this extrapolation strategy may have problems if hazard rates change substantially in the long term. In the CULPRIT-SHOCK trial, mortality risk is higher in the MV-PCI arm. However, after 30-day follow-up, the pattern seems to change with non-statistically significant differences in mortality between arms favouring the MV-PCI. A change in mortality rates comparing short to long term has also been found in non-randomised studies [41, 42]. Based on this evidence, and following guidelines, two main modelling decisions were taken to improve clinical and external validity of extrapolation of mortality risk [25]. First, only the period after 30-day follow-up have been considered for the estimation of death risk. And second, mortality was assumed to change proportionally to life tables rates to avoid unsustainable survival rates in the long-term.

Another feature of the results is the uncertainty about cost effectiveness. A substantial probability for MV-PCI being more cost-effective remains, ranging 35–33% for thresholds 30,000–50,000 €/QALY (lifelong model). Uncertainty comes from the estimated parameters; especially long-term probability of death, heart failure and MACE. For example, long-term mortality rate, extrapolated from 30-day to 1-year follow-up data, was not statistically significantly different in CO-PCI compared to MV-PCI. However, the inclusion of this estimate adds uncertainty to the results. Even more, the long-term analysis comes at a higher uncertainty because of the extrapolation exercise. Two scenarios were considered to illustrate the statistical uncertainty under different extrapolation hypothesis (see Online Appendix). In scenario 1, we assumed no differences between arms in risk of death in the long term. The consequence was a > 75% of CO-PCI being cost-effective for a threshold of 30,000€/QALY. In scenario 2, we assumed no differences between arms in risk of death, heart failure and MACE in the long term. The same probability of CO-PCI being cost-effective increased to > 90%.

The cost-effectiveness impact of the two revascularization approaches in multivessel disease complicated by cardiogenic shock is based on an intention to treat principle according to good research practice [43]. In this sense, crossovers were possible due to medical decisions or technical reasons. For example, 43 (12.5%) patients in the CO-PCI arm undertook immediate multivessel PCI. On the other hand, 32 (9.4%) patients allocated to the MV-PCI were not treated with immediate PCI of non-culprit lesions. In addition, the generalisability of the results relies on whether revascularisation procedures are followed in line with the CULPRIT-SHOCK definitions. For example, some studies may consider different definitions of complete multivessel revascularization [44].

## Conclusion

On average, results favour the CO-PCI strategy as the most-cost effective strategy if the monetary value of a QALY is higher than €7010. However, substantial uncertainty remains even at a threshold of €30,000 per QALY, mainly due to extrapolation of long-term parameters from the trial data. This uncertainty would be widely reduced if we were certain that there was no direct treatment effect after the trial period in key parameters like long term mortality, heart failure or MACE. Finally, results show that cost-effectiveness improves with age of the patient and for the non-diabetes groups.

## Electronic supplementary material

Below is the link to the electronic supplementary material.

Supplementary file1 (DOCX 1745 kb)

## Footnotes

1 The British National Formulary (BNF) was used where medication prices were not available at the DIMDI.

2 Patients with more than 365 days of hospitalisation or more than 7 h of fluoroscopy were considered outliers. Less than 1% of patients met one of these two criteria.
