# Supplementary material for: Economic evaluation of culprit lesion only PCI vs. immediate multivessel PCI in acute myocardial infarction complicated by cardiogenic shock: the CULPRIT-SHOCK trial
Source: Eur J Health Econ. 2020 Oct 7;21(8):1197–209. doi: 10.1007/s10198-020-01235-3 (PMC7561561; doi:10.1007/s10198-020-01235-3)
Supplement: Supplementary file 1 — Supplementary file1 (DOCX 1745 kb) [file 10198_2020_1235_MOESM1_ESM.docx]

**Appendix 1: Unit costs**

| Table A1. Unit costs. 2018 Euros | | | | |
| --- | --- | --- | --- | --- |
| **Type of cost** | **Unit cost (€)** | **Source** | **Codes** | **Description/Explanation** |
| PCI | 3,247.47 | G-DRG 2018 | *F24A, F52A, F56A, F58A* | Average used. Hospitalization and medication costs excluded |
| Renal rep. therapy: |  |  |  |  |
| Intermittent dialysis | *230.63* | G-DRG 2018 | *ZE01.01* |  |
| Hemofiltration | *954.53* | G-DRG 2018 | *ZE119.02 (8-853.14)* |  |
| Continuous dialysis | *812.38* | G-DRG 2018 | *ZE120.02 (8-854.61)* |  |
| Hemodiafiltration | *932.68* | G-DRG 2018 | *ZE121.02 (8-855.71)* |  |
| Stents | 68.75 | G-DRG 2018 | ZE101.01 | Drug eluting stent (DES) unit cost used |
| Angiography | 470.05 | Literature ^1^ |  |  |
| ICD | 2,795.65 | G-DRG 2018 | *F01A, F01B, F01C, F01D, F01E, F01F* | Average used. Only theatre and anaesthesia costs considered |
| ECMO | 9,632.74 | Literature ^2^ |  | Three days ECMO considered |
| IABP | 1,267.70 | Literature ^2^ |  |  |
| LVAD | 43,129.19 | Literature ^2^ |  |  |
| Heart Transplant | 19,909.00 | G-DRG 2018 | *A05A, A05B* | Average used. Only theatre and anaesthesia costs considered |
| Investigations: |  |  |  |  |
| MRI | 391.70 | Literature ^3^ |  |  |
| Scintigraphy | 91.57 | Literature ^3^ |  |  |
| Stress-Echocardiogram | 31.54 | Literature ^4^ |  |  |
| ICU days | 960.44 | Literature ^5^ |  |  |
| Normal ward/inpatient days | 285.89 | Literature ^5^ |  |  |
| Emergency room visit | 391.26 | WHO^6^ |  |  |
| Labour cost (hour) | 34.55 | DeStatis |  |  |
| Renal failure (within trial cost) | 64810.70 | Literature ^7^ |  |  |
| Medications (€/mg): |  |  |  |  |
| Aspirin | 0.000474 | DIMDI |  | Dose assumed: 75 mg/day |
| Clopidogrel | 0.037140 | DIMDI |  | Dose assumed: 300 mg/day |
| Prasugrel | 0.134498 | BNF |  | Dose assumed: 10 mg/day |
| Ticagrelor | 0.007910 | Literature |  | Dose assumed: 180 mg/day |
| GP IIb/IIIa-Inhibitors | 0.203500 | BNF |  | Dose assumed: 921.78 mg in total |
| UF Heparin | 0.000151 | DIMDI |  | Dose assumed: 34.56 mg in total |
| LMW Heparin | 0.094896 | DIMDI |  | Dose assumed: 160 mg/day |
| Bivalirudin | 0.554280 | BNF |  | Dose assumed: 900 mg in total |
| Fondaparinux | 0.497189 | BNF |  | Dose assumed: 2.5 mg/day |
| Beta-Blocker | 0.089733 | DIMDI |  | Dose assumed: 100 mg/day |
| ACE-Inhibitor/AT-II-Antagonist | 0.010024 | DIMDI |  | Dose assumed: 150 mg/day |
| Statins | 0.048979 | DIMDI |  | Dose assumed: 80 mg/day |
| Catecholamine Therapy | 0.017895 | BNF |  | Dose assumed: 403.2 mg in total |
| Calcium-Antagonist | 0.017800 | DIMDI |  | Dose assumed: 7 mg/day |
| Aldosterone-Antagonist | *0.024162* | BNF |  | Dose assumed: 50 mg/day |
| Diuretics | *0.003281* | BNF |  | Dose assumed: 2.5 mg/day |
| Edoxaban | *0.023095* | BNF |  | Dose assumed: 60 mg/day |
| Apixaban | *0.300895* | BNF |  | Dose assumed: 10 mg/day |
| Rivaroxaban | *0.285058* | BNF |  | Dose assumed: 5 mg/day |
| Dabigatran | *0.004487* | BNF |  | Dose assumed: 260 mg/day |
| Vitamin-K-Antagonists | *0.261304* | BNF |  | Dose assumed: 7.5 mg/day |
| Note 1. Abbreviations: G-DRG 2018 – Diagnosis related group of the Institute for the Hospital Remuneration System (InEK); WHO – World health organization; DeStatis - Federal Statistical Office; DIMDI – German Institute of Medical Documentation and Information; BNF – British National Formulary.  Note 2. All costs were converted to 2018 Euros using the consumer price index (CPI) and purchasing power parity (PPP) when cost expressed in other currency. | | | | |

**Appendix 2. Estimation of long-term Markov probabilities**

Parametric survival analysis was applied to the within trial data (up to 1 year from randomisation) to estimate long-term probability of events in the Markov model. The next parametric models were considered:

- Exponential
- Gompertz
- Weibull
- Loglogistic
- Lognormal

The best-fit model, according to the Akaike’s information criterion (AIC) and Bayesian information criterion (BIC), for each separate event was chosen to populate the decision model. Visual inspection analysis was performed by comparing the best-fit parametric survival functions to Kaplan-Meier curves.

In the next sections, we explain the process for computing the probability of each event.

**Mortality**

Death is the event being analysed. Descriptive analysis showed a changing pattern for the first 30 days compared to the 30-days to 1-year after randomisation period. First, mortality probability is about four times higher in the former period (see table 1 in the main text). Second, the relative risk for CO-PCI vs. MV-PCI changes in the latter with respect to the former, from 0.84 (p-value = 0.033) to 1.09 (p-value = 0.777) respectively. Based on this information, we decided to use the data for the 30-days to 1-year period to estimate the arm effect and extrapolate mortality in the long-term. The explanatory variables included in the model were: trial arm, subgroup characteristics (age, gender, diabetes or not) and health state (A&S, renal failure, heart failure and MACE).

To obtain a hazard risk conditional on health state, the dataset was expanded for those patients that experienced an event like renal replacement therapy, rehospitalisation for heart failure, or MACE (urgent revascularisation, stroke and MI). Each patient could have as many time spans as health states have experienced over the period considered. This way we could estimate the incremental effect of having the additional condition.

The best-fit model was Weibull (see Table A2). In Figure A1, Weibull model fit and Kaplan-Meier curves are compared by arms (including trial arms as the only explanatory variables in the model) for visual inspection.

The Weibull survival function is:

| $S\left( t \right)=exp(-\lambda t^{p})$. | (A1) |
| --- | --- |

Where *t* is time (in days) from 30-day follow-up. The estimates of parameters $\lambda$ and $p$ (in logarithm) are shown in Table A3.

Death transition probability for the first cycle in the Markov model is computed as:

| $tp\left( t \right)=1-S\left( t+m \right)/S\left( t \right)$. | (A2) |
| --- | --- |

Where *t* is the stating time-point and *m* is the length of the cycle. The death transition probabilities are assumed to change proportionally to death rates in life tables (see Table A4) for the following Markov cycles.

| Table A2. Fit of parametric models. Mortality | | |
| --- | --- | --- |
| **Model** | **AIC** | **BIC** |
| Exponential | 393.69 | 430.53 |
| Gompertz | 385.68 | 426.61 |
| Weibull | 381.43 | 422.36 |
| Loglogistic | 382.85 | 423.79 |
| Lognormal | 387.75 | 428.68 |
| Note. Akaike’s information criterion (AIC) and Bayesian information criterion (BIC) considered | | |

Figure A1. Weibull survival curve vs. Kaplan-Meier curves by arm

| Table A3. Estimates of parametric survival model. Weibull | | |
| --- | --- | --- |
|  | Coef. | p-value |
| Log($\lambda$): |  |  |
| CO-PCI | 0.019 | 0.953 |
|  |  |  |
| Health state (ref. A&S): |  |  |
| HF | 0.630 | 0.328 |
| MACE | 0.528 | 0.181 |
| RF | 0.857 | 0.062 |
| Subgroup variables |  |  |
| Age (ref. <50): |  |  |
| 50-75 | 0.407 | 0.693 |
| >75 | 1.555 | 0.135 |
| Male | 0.576 | 0.179 |
| Diabetes | 0.820 | 0.010 |
| Cons | -7.152 | 0.000 |
| Log($p$): | -0.578 | 0.000 |
| *N* | 434 |  |
| Note. The constant of the model represents a female patient, in the A&S state, younger than 50 and with no diabetes. | | |

| Table A4. Life table. World health organization. Germany 2016 | | |
| --- | --- | --- |
|  | nMx - death rate | |
| Age interval | Male | Female |
| 40-44 years | 0.001 | 0.001 |
| 45-49 years | 0.002 | 0.001 |
| 50-54 years | 0.004 | 0.002 |
| 55-59 years | 0.007 | 0.004 |
| 60-64 years | 0.013 | 0.006 |
| 65-69 years | 0.016 | 0.009 |
| 70-74 years | 0.027 | 0.015 |
| 75-79 years | 0.041 | 0.024 |
| 80-84 years | 0.076 | 0.053 |
| 85+ years | 0.177 | 0.154 |
| Note 1. nMx - is directly provided by World Health Organization as the age-specific death rate.  Note 2. Monthly death probabilities were computed as 1-(1-nMx)^1/12^  Note 3. For the basecase analysis, the average rate for males and females are used. The patient is assumed to enter the model at the sample average age. Subgroup analysis will use life table information specific to each subgroup. | | |

**Renal failure**

No new patients required renal replacement therapy after the first 19 days from randomisation (see Kaplan-Meier curves in Figure A2). For the long-term, it was assumed that the risk of renal failure is zero or not quantitatively relevant for both arms.

Figure A2. Kaplan-Meier renal-failure-free survival curves by arm

**Heart failure**

Heart failure is the event being analysed. The explanatory variables included in the model were trial arm and subgroup characteristics (age, gender, diabetes or not). The best-fit model was Exponential (see Table A5). In Figure A3, Exponential model fit and Kaplan-Meier curves are compared by arms (including trial arms as the only explanatory variables in the model) for visual inspection.

The exponential survival function is:

| $S\left( t \right)=exp(-\lambda t)$. | (A3) |
| --- | --- |

Where *t* is time (in days) from randomisation. The estimates of parameter $\lambda$ (in logarithm) is shown in Table A6.

Heart failure transition probability for the Markov model is constant over time and computed as:

| $tp\left( t \right)=1-\frac{S\left( t+m \right)}{S\left( t \right)}=1-exp(-\lambda m)$ | (A4) |
| --- | --- |

where *m* is the length of the cycle.

| Table A5. Fit of parametric models. Heart failure | | |
| --- | --- | --- |
| **Model** | **AIC** | **BIC** |
| Exponential | 213.42 | 240.59 |
| Gompertz | 214.62 | 246.32 |
| Weibull | 215.25 | 246.95 |
| Loglogistic | 215.42 | 247.13 |
| Lognormal | 215.49 | 247.20 |
| Note. Akaike’s information criterion (AIC) and Bayesian information criterion (BIC) considered | | |

Figure A3. Exponential heart-failure-free survival curve vs. Kaplan-Meier curves by arm

| Table A6. Estimates of parametric heart-failure-free survival model. Exponential | | |
| --- | --- | --- |
|  | Coef. | p-value |
| Log($\lambda$): |  |  |
| CO-PCI | 1.431 | 0.010 |
|  |  |  |
| Subgroup variables |  |  |
| Age (years) | 0.034 | 0.111 |
| Male | -0.193 | 0.693 |
| Diabetes | 0.351 | 0.442 |
| Cons | -10.960 | 0.000 |
| *N* | 685 |  |
| Note. The constant of the model represents a female patient, age 68 and with no diabetes. | | |

**MACE**

MACE is the event being analysed. The explanatory variables included in the model were trial arm and subgroup characteristics (age, gender, diabetes or not). The best-fit model was Lognormal (see Table A7). In Figure A4, Lognormal model fit and Kaplan-Meier curves are compared by arms (including trial arms as the only explanatory variables in the model) for visual inspection.

The Lognormal survival function is:

| $S\left( t \right)=1-\Phi\left\{ \frac{\log\left( t \right)-\mu}{\sigma} \right\}$. | (A5) |
| --- | --- |

Where *t* is time (in days) from randomisation. The standard normal cumulative distribution is $\Phi$. The estimates of parameters $\mu$ and $\sigma$ (in logarithm) are shown in Table A8.

MACE transition probability for the Markov model at moment $t$ is computed as:

| $tp\left( t \right)=1-\frac{S\left( t+m \right)}{S\left( t \right)}$ | (A6) |
| --- | --- |

where *m* is the length of the cycle.

| Table A7. Fit of parametric models. MACE | | |
| --- | --- | --- |
| **Model** | **AIC** | **BIC** |
| Exponential | 1073.517 | 1100.693 |
| Gompertz | 979.444 | 1011.15 |
| Weibull | 923.78 | 955.486 |
| Loglogistic | 922.23 | 953.941 |
| Lognormal | 919.28 | 950.993 |
| Note. Akaike’s information criterion (AIC) and Bayesian information criterion (BIC) considered | | |

Figure A4. Lognormal MACE-free survival vs. Kaplan-Meier curves by arm

| Table A8. Estimates of parametric MACE-free survival model. Lognormal | | |
| --- | --- | --- |
|  | Coef. | p-value |
| $\mu$: |  |  |
| CO-PCI | -1.053 | 0.048 |
| Subgroup variables |  |  |
| Age (ref. <50): |  |  |
| 50-75 | 0.184 | 0.869 |
| >75 | 0.959 | 0.432 |
| Male | 0.676 | 0.275 |
| Diabetes | 0.187 | 0.748 |
| Cons | 9.315 | 0.000 |
| Log($\sigma$) | 1.457 | 0.000 |
| *N* | 685 |  |
| Note. The constant of the model represents a female patient, age<50 and with no diabetes. | | |

**Appendix 3. Base-case model parameters**

| Table A9. Parameters of the model. Basecase | | | | |
| --- | --- | --- | --- | --- |
| **Parameter** | **Description** | **Mean/α** | **SD/β** | **Distribution** |
| Probabilities for decision tree: |  |  |  |  |
| p_death30d_MV | Probability of death 30 days after MV-PCI | 176 | 166 | Beta |
| p_death30d_1y_MV | Conditional probability of death between 30 days and 1 year after MV-PCI | 18 | 148 | Beta |
| p_mace30d_1y _MV | Conditional probability of MACE between 30 days and 1 year after MV-PCI | 33 | 133 | Beta |
| p_renal30d_1y _MV | Conditional probability of renal failure between 30 days and 1 year after MV-PCI | 9 | 157 | Beta |
| p_heart30d_1y _MV | Conditional probability of heart failure between 30 days and 1 year after MV-PCI | 4 | 162 | Beta |
| rr_death30d_CO | Relative Risk of death 30 days after CO-PCI | .8416 | .0681 | Lognormal |
| rr_death30d_1y_CO | Relative Risk of death between 30 days and 1 year after CO-PCI | 1.087 | .3224 | Lognormal |
| rr_mace_CO | Relative Risk of MACE 1 year after CO-PCI | 2.2442 | .3928 | Lognormal |
| rr_renal_CO | Relative Risk of renal failure 1 year after CO-PCI | .6621 | .3262 | Lognormal |
| rr_heart_CO | Relative Risk of heart failure 1 year after CO-PCI | 2.5538 | 1.4495 | Lognormal |
| Transition probabilities for Markov model: | |  |  |  |
| p_death | Long term monthly probability of death (conditional on health state and arm) | See survival analysis in appendix | | |
| p_mace | Long term monthly probability of MACE from any state (conditional on arm) | See survival analysis in appendix | | |
| p_heart | Long term monthly probability of heart failure (conditional on arm) | See survival analysis in appendix | | |
| p_renal | Long term monthly probability of renal failure | See survival analysis in appendix | | |
| Cost parameters for decision tree: | | | | |
| treat_MV | Treatment costs for multivessel revascularization | 24530.6 | 1464.1 | Gamma |
| treat_CO | Treatment costs for culprit only revascularization | 25371.4 | 1411.6 | Gamma |
| Cost parameters for Markov model: |  |  |  |  |
| cost_MACE | One off cost for MACE | 92.5 | 636.1 | Gamma |
| cost_renal | Monthly cost for renal failure | 65.9 | 186.0 | Gamma |
| cost_heart | Monthly cost for heart failure | 320.4 | 192.2 | Gamma |
| cost_death | One off cost for death (if dies before 65) | 16584 | - | - |
| Utilities of health states: |  |  |  |  |
| u_stable | Utility for patient in *A&S* | .78493 | .040971 | Beta |
| u_MACE | Utility increment for MACE | .0358972 | .023686 | Beta |
| u_renal | Utility decrement for renal failure | .0849644 | .060618 | Beta |
| u_heart | Utility decrement for heart failure | .0508218 | .048408 | Beta |
| u_MACE_long | Utility increment for MACE for the Markov model | .0103975 | .027683 | Beta |
| Note 1. One off cost for death is considered assuming a friction cost of 3 months (60 working days, 8hrs/day) | | | | |
|  | | | | |

**Appendix 4. Analysis of missing data**

| **Table A10. Missing values for hospitalization/emergency room** | | | | | |
| --- | --- | --- | --- | --- | --- |
| Type of hospital | Total missing | Missing converted to zero | Missing replaced by average | Average for death patients | Average for patients alive |
| Health State: |  |  |  |  |  |
| *ICU (days)* | 46 | 41 | 5 | 4.20 | 11.00 |
| *Normal ward (days)* | 116 | 53 | 63 | .01 | 10.49 |
| *Inpatient days (6m FU)* | 367 | 325 | 42 | .039 | 3.94 |
| *Inpatient days (12m FU)* | 427 | 345 | 82 | 0 | 2.67 |
| Emergency room visits (30day FU) | 335 | 216 | 119 | .036 | .214 |
| Emergency room visits (6m FU) | 364 | 325 | 39 | .04 | .24 |
| Emergency room visits (12m FU) | 377 | 349 | 28 | .008 | .073 |
| Note 1. Missing converted to zero are those patients that died before they could have any hospitalization at that time.  Note 2. Average for deaths patients took the average those patients that died before each FU. Average for patients alive took the average for those patients alive after each FU. In case of ICU and normal ward, patients were grouped according to their survival status at 30 days after randomisation. | | | | | |

**Appendix 5. Subgroup analyses**

| Table A11. Parameters of the model. Subgroups | | | | | | | | | | | | | | | | | |
| --- | --- | --- | --- | --- | --- | --- | --- | --- | --- | --- | --- | --- | --- | --- | --- | --- | --- |
|  | *Age <50* | | *Age 50 – 75* | | *Age >75* | | *Female* | | *Male* | | *No diabetes* | | *Diabetes* | | **Distribution** | |  |
| **Parameter** | **Mean**  **(α)** | **SD**  **(β)** | **Mean**  **(α)** | **SD**  **(β)** | **Mean**  **(α)** | **SD**  **(β)** | **Mean**  **(α)** | **SD**  **(β)** | **Mean**  **(α)** | **SD**  **(β)** | **Mean**  **(α)** | **SD**  **(β)** | **Mean**  **(α)** | **SD**  **(β)** |  |  |  |
| Probabilities for decision tree: | | | | | | | | | | | | | | | | | |
| p_death30d_MV | 3 | 13 | 104 | 123 | 69 | 30 | 41 | 34 | 135 | 132 | 109 | 110 | 60 | 56 | Beta |  |  |
| p_death30d_1y_MV | 0 | 13 | 9 | 114 | 9 | 21 | 3 | 31 | 15 | 117 | 7 | 103 | 11 | 45 | Beta |  |  |
| p_mace30d_1y _MV | 1 | 12 | 27 | 96 | 5 | 25 | 10 | 24 | 23 | 109 | 21 | 89 | 3 | 53 | Beta |  |  |
| p_renal30d_1y _MV | 0 | 13 | 8 | 115 | 1 | 29 | 0 | 34 | 9 | 123 | 6 | 104 | 12 | 44 | Beta |  |  |
| p_heart30d_1y _MV | 0 | 13 | 4 | 119 | 0 | 30 | 1 | 33 | 3 | 129 | 3 | 107 | 1 | 55 | Beta |  |  |
| rr_death30d_CO | 0.842 | 0.068 | 0.842 | 0.068 | 0.842 | 0.068 | 0.842 | 0.068 | 0.842 | 0.068 | 0.842 | 0.068 | 0.842 | 0.068 | Lognormal |  |  |
| rr_death30d_1y_CO | 1.087 | 0.322 | 1.087 | 0.322 | 1.087 | 0.322 | 1.087 | 0.322 | 1.087 | 0.322 | 1.087 | 0.322 | 1.087 | 0.322 | Lognormal |  |  |
| rr_mace_CO | 2.244 | 0.393 | 2.244 | 0.393 | 2.244 | 0.393 | 2.244 | 0.393 | 2.244 | 0.393 | 2.244 | 0.393 | 2.244 | 0.393 | Lognormal |  |  |
| rr_renal_CO | 0.662 | 0.326 | 0.662 | 0.326 | 0.662 | 0.326 | 0.662 | 0.326 | 0.662 | 0.326 | 0.662 | 0.326 | 0.662 | 0.326 | Lognormal |  |  |
| rr_heart_CO | 2.554 | 1.450 | 2.554 | 1.450 | 2.554 | 1.450 | 2.554 | 1.450 | 2.554 | 1.450 | 2.554 | 1.450 | 2.554 | 1.450 | Lognormal |  |  |
| Transition probabilities for Markov model: | | | | | | | | | | | | | | | | | |
| p_death | See survival analysis in appendix | | | | | | | | | | | | | | | | |
| p_mace | See survival analysis in appendix | | | | | | | | | | | | | | | | |
| p_heart | See survival analysis in appendix | | | | | | | | | | | | | | | | |
| p_renal | See survival analysis in appendix | | | | | | | | | | | | | | | | |
| Cost parameters for decision tree: | | | | | | | | | | | | | | | |  |  |
| treat_MV | 31930.8 | 4686.3 | 26971.7 | 1852.2 | 17691.6 | 2466.5 | 18210.4 | 2394.9 | 26360.0 | 1743.3 | 24590.9 | 1811.0 | 24392.0 | 2559.4 | Gamma |  |  |
| treat_CO | 39346.4 | 6602.4 | 28753.1 | 1839.1 | 17234.6 | 2125.1 | 19065.6 | 2552.7 | 27463.7 | 1659.4 | 25033.5 | 1690.9 | 26236.4 | 2719.1 | Gamma |  |  |
| Cost parameters for Markov model: | | | | | | | | | | | | | | | |  |  |
| cost_MACE | 92.5 | 636.1 | 92.5 | 636.1 | 92.5 | 636.1 | 92.5 | 636.1 | 92.5 | 636.1 | 92.5 | 636.1 | 92.5 | 636.1 | Gamma |  |  |
| cost_renal | 65.9 | 186.0 | 65.9 | 186.0 | 65.9 | 186.0 | 65.9 | 186.0 | 65.9 | 186.0 | 65.9 | 186.0 | 65.9 | 186.0 | Gamma |  |  |
| cost_heart | 320.4 | 192.2 | 320.4 | 192.2 | 320.4 | 192.2 | 320.4 | 192.2 | 320.4 | 192.2 | 320.4 | 192.2 | 320.4 | 192.2 | Gamma |  |  |
| cost_death | 16584 | - | 16584 | - |  |  | 16584 | - | 16584 | - | 16584 | - | 16584 | - | - |  |  |
| Utilities of health states: | | | | | | | | | | | | | | | |  |  |
| u_stable | 0.8200 | 0.0615 | 0.8036 | 0.0374 | 0.7044 | 0.0463 | 0.7392 | 0.0490 | 0.7959 | 0.0391 | 0.8001 | 0.0401 | 0.7485 | 0.0431 | Beta |  |  |
| u_MACE | 0.0359 | 0.0237 | 0.0359 | 0.0237 | 0.0359 | 0.0237 | 0.0359 | 0.0237 | 0.0359 | 0.0237 | 0.0359 | 0.0237 | 0.0359 | 0.0237 | Beta |  |  |
| u_renal | 0.0850 | 0.0606 | 0.0850 | 0.0606 | 0.0850 | 0.0606 | 0.0850 | 0.0606 | 0.0850 | 0.0606 | 0.0850 | 0.0606 | 0.0850 | 0.0606 | Beta |  |  |
| u_heart | 0.0508 | 0.0484 | 0.0508 | 0.0484 | 0.0508 | 0.0484 | 0.0508 | 0.0484 | 0.0508 | 0.0484 | 0.0508 | 0.0484 | 0.0508 | 0.0484 | Beta |  |  |
| u_MACE_long | 0.0104 | 0.0277 | 0.0104 | 0.0277 | 0.0104 | 0.0277 | 0.0104 | 0.0277 | 0.0104 | 0.0277 | 0.0104 | 0.0277 | 0.0104 | 0.0277 | Beta |  |  |
| Note 1. One off cost for death is considered assuming a friction cost of 3 months (60 working days, 8hrs/day) | | | | | | | | | | | | | | | | | |


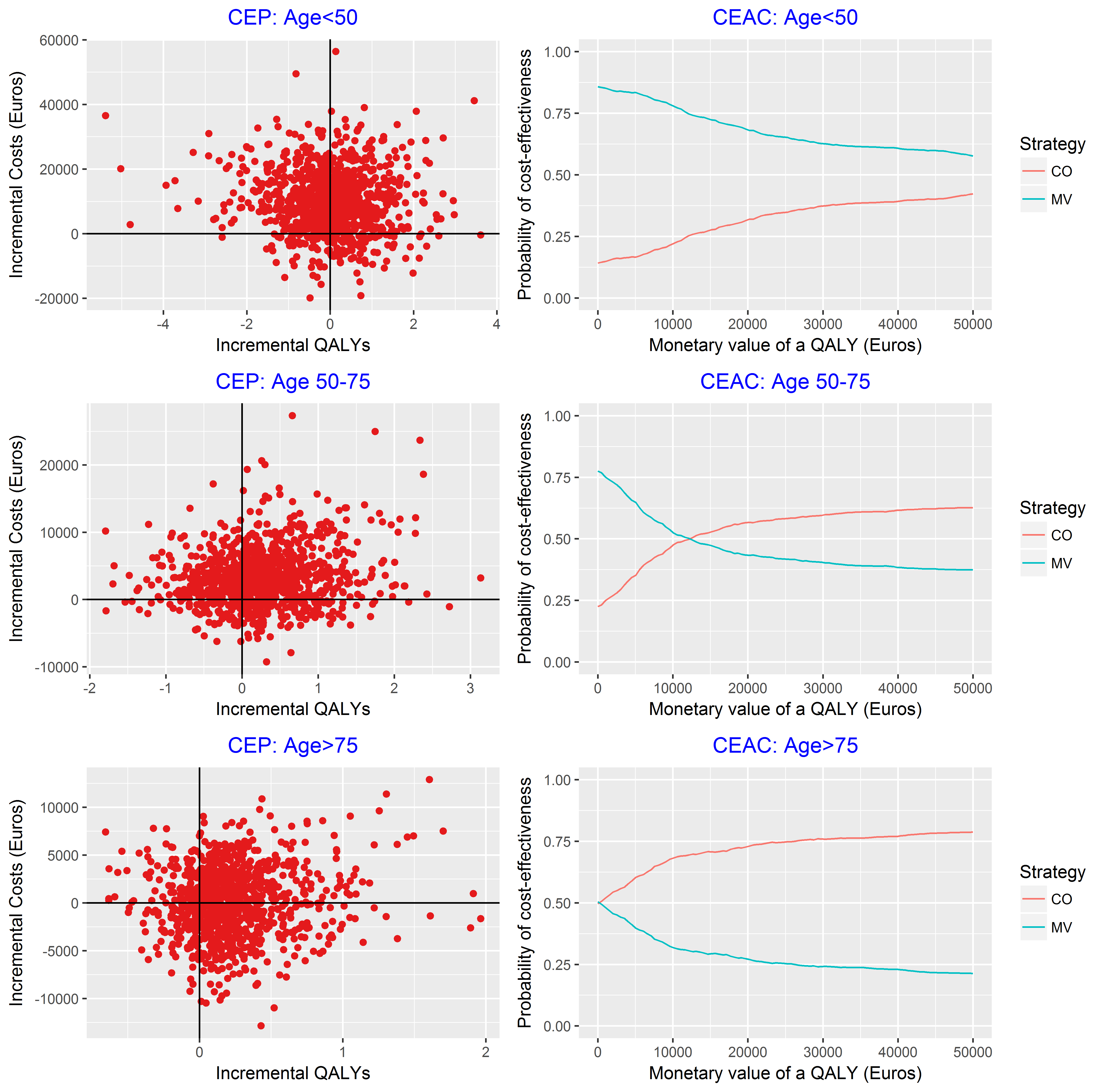


Figure A5. Subgroup analysis. Age


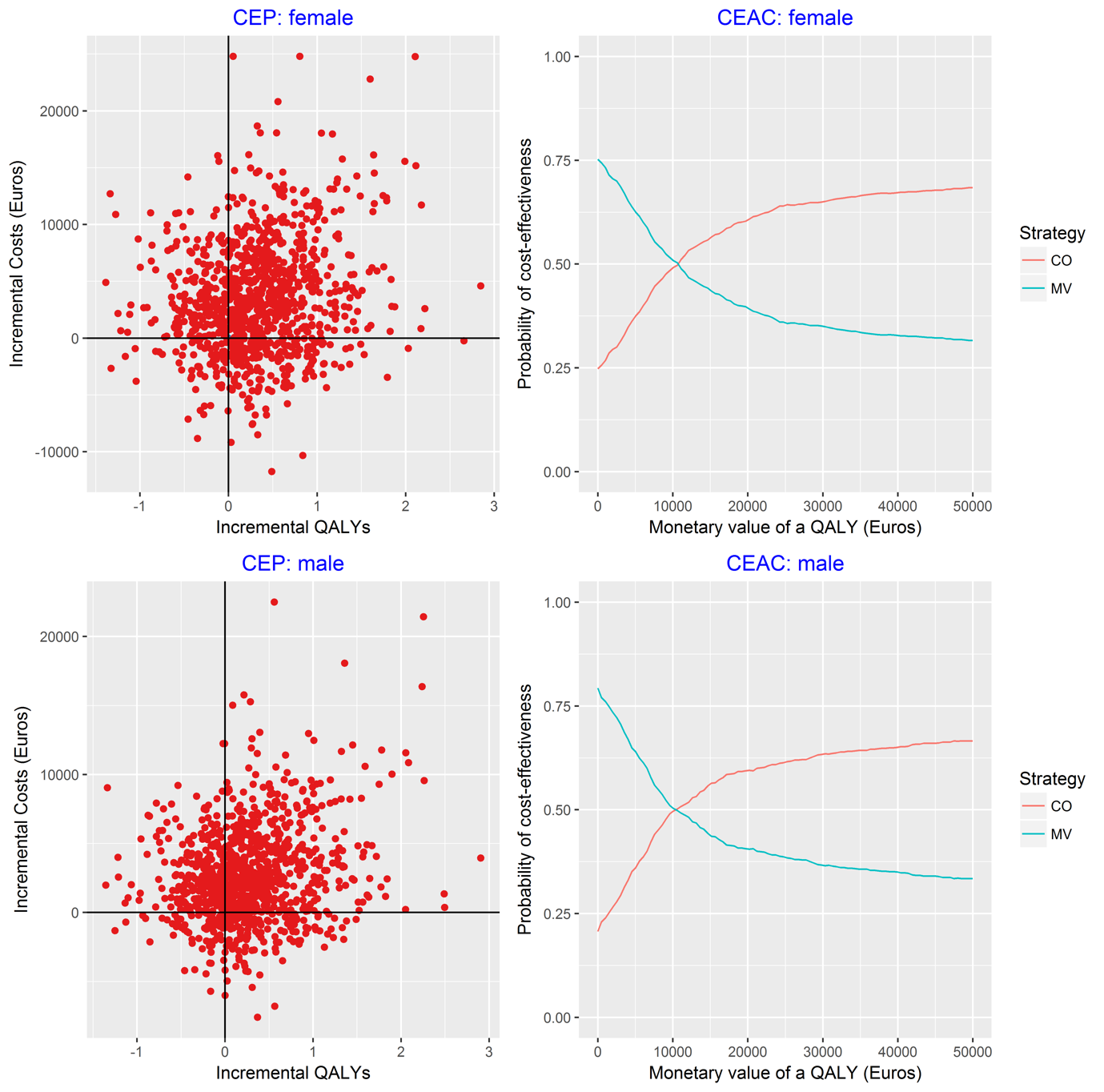


Figure A6. Subgroup analysis. Gender


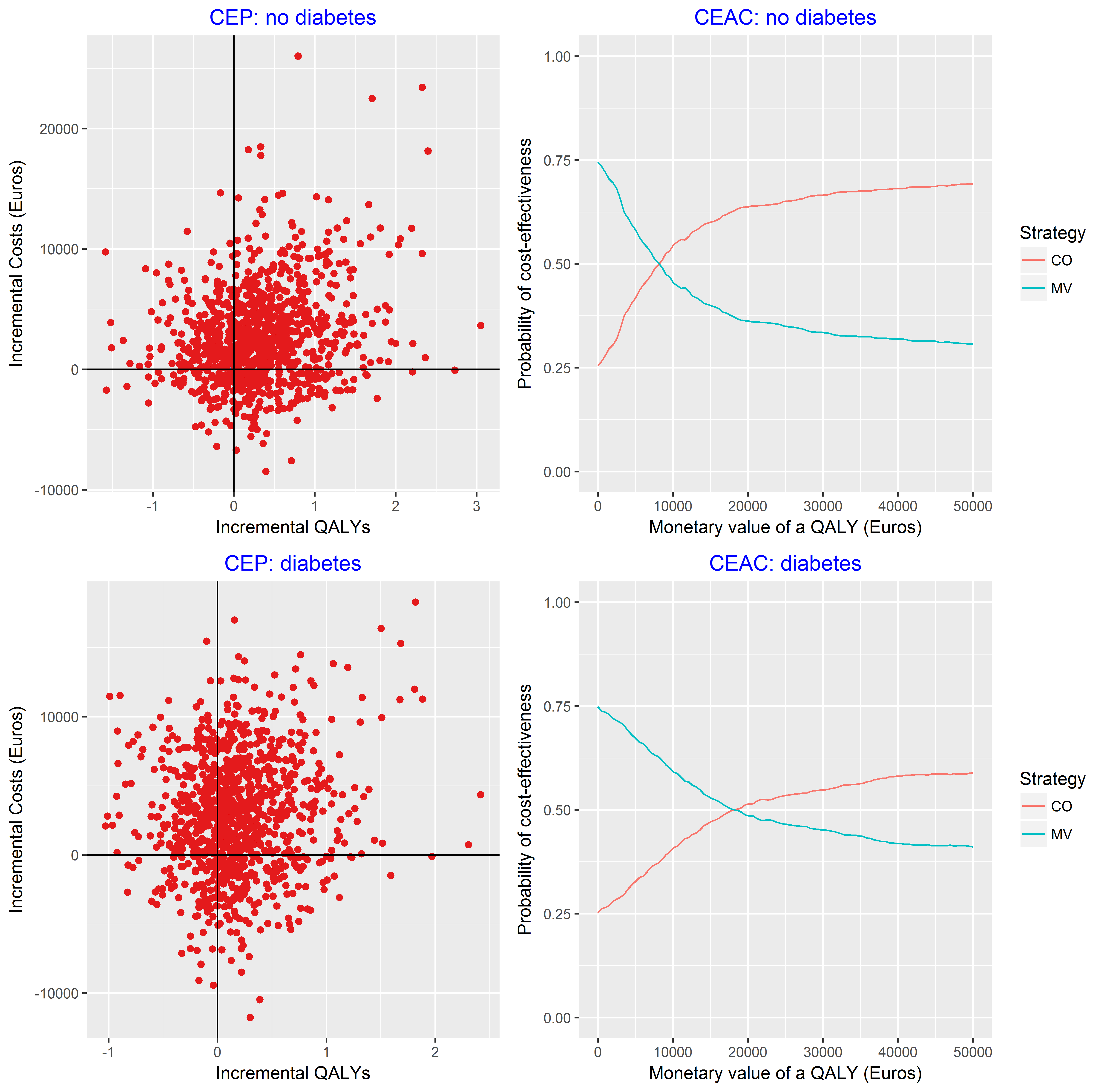


Figure A7. Subgroup analysis. Diabetes

**Appendix 6.** **Scenario analyses**

Scenario 1. Assuming no differences between CO-PCI and MV-PCI arms in the long-term risk of death, i.e. CO-PCI coefficient in Table A3 is assumed zero.

| Table A12. Lifelong CUA results: costs, effects and ICERs. Scenario 1. | | | | | | |
| --- | --- | --- | --- | --- | --- | --- |
|  | Costs (€/patient) | | QALYs (per patient) | | ICER (€/QALY) | |
| Intervention strategies | Mean | [95% CI] | Mean | [95% CI] | Mean | [95% CI] |
|  |  |  |  | |  | |
| *CO-PCI* | 27200 | [23500, 38300] | 2.96 | [1.10, 5.36] |  |  |
| *MV-PCI* | 25100 | [22400, 29700] | 2.64 | [0.97, 4.84] |  |  |
| *CO-PCI vs. MV-PCI* | 2080 | [-2350, 10900] | 0.32 | [-.47, 1.17] | 6550 | [-94500, 74800] |
|  | | | | | | |


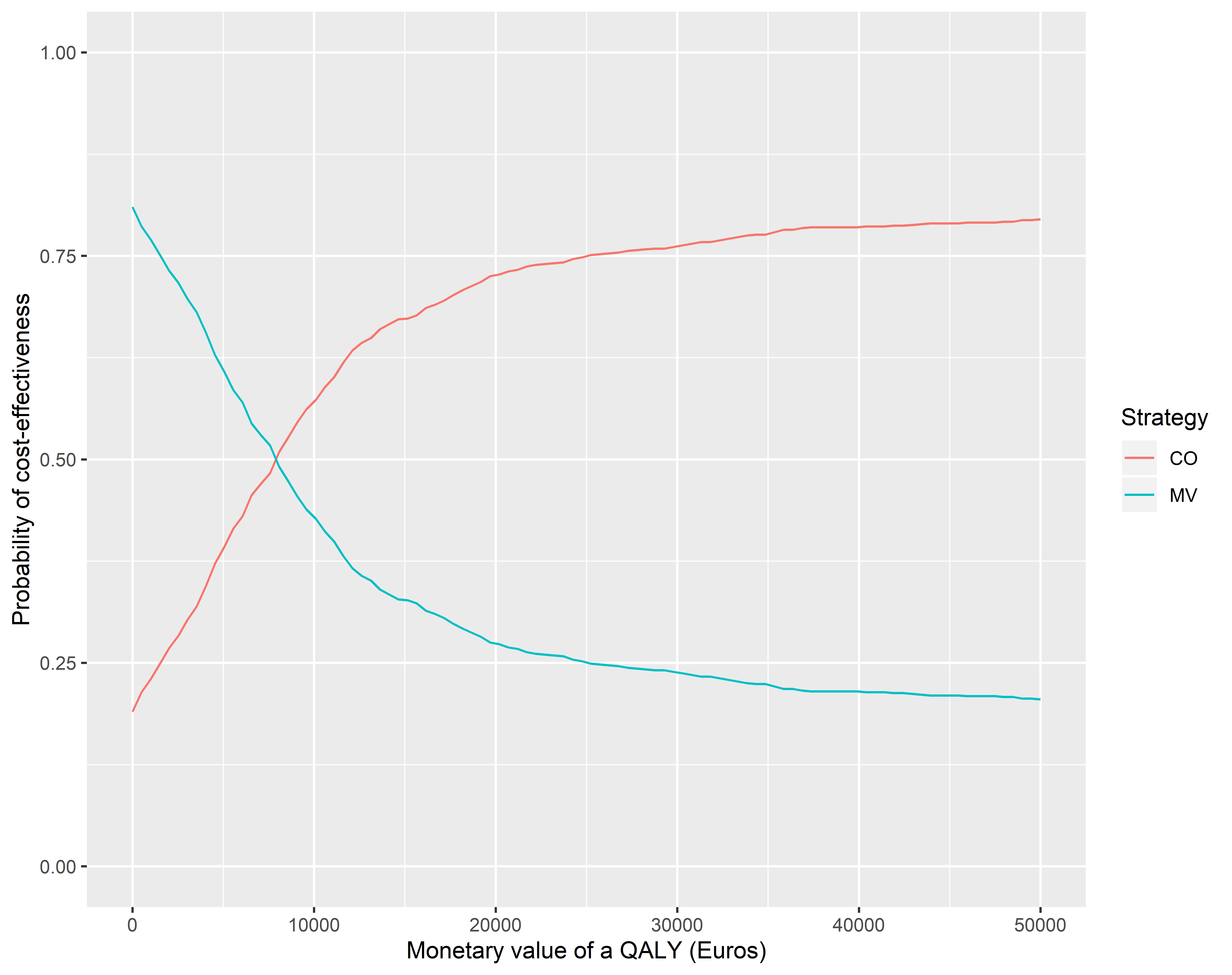


Figure A8. CEAC. Lifelong CUA scenario 1.

Scenario 2. Assuming no differences between CO-PCI and MV-PCI arms in the long-term risk of death, heart failure and MACE, i.e. CO-PCI coefficients in Table A3, A6 and A8 are assumed zero.

| Table A13. Lifelong CUA results: costs, effects and ICERs. Scenario 2. | | | | | | |
| --- | --- | --- | --- | --- | --- | --- |
|  | Costs (€/patient) | | QALYs (per patient) | | ICER (€/QALY) | |
| Intervention strategies | Mean | [95% CI] | Mean | [95% CI] | Mean | [95% CI] |
|  |  |  |  | |  | |
| *CO-PCI* | 26400 | [23200, 32200] | 3.04 | [1.13, 5.56] |  |  |
| *MV-PCI* | 25100 | [22400, 29700] | 2.64 | [0.97, 4.84] |  |  |
| *CO-PCI vs. MV-PCI* | 1300 | [-2890, 5940] | 0.40 | [-.07, 1.10] | 3250 | [-33700, 47800] |
|  | | | | | | |


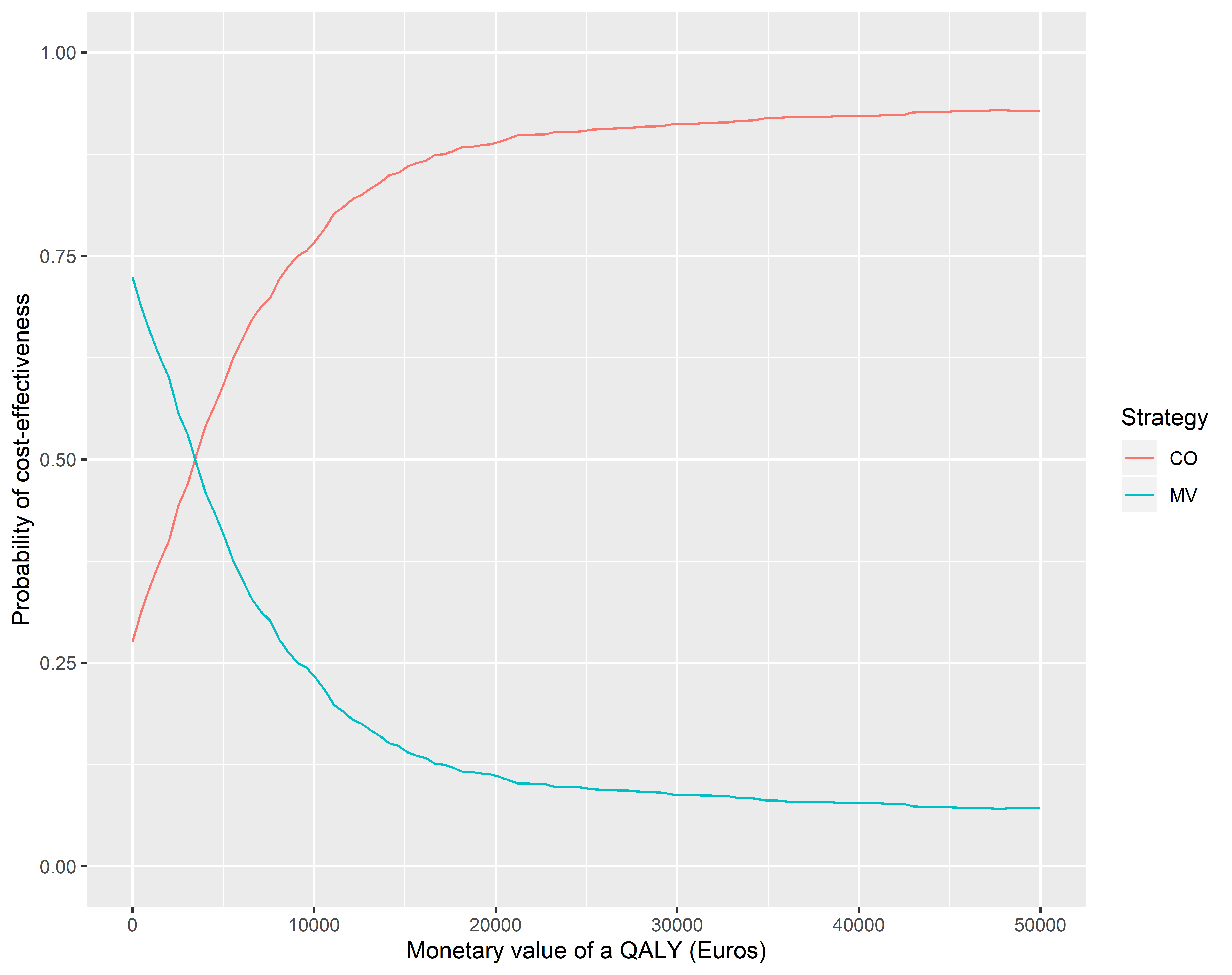


Figure A9. CEAC. Lifelong CUA scenario 2.

**References**

1. Moschetti K, Muzzarelli S, Pinget C, et al. Cost evaluation of cardiovascular magnetic resonance versus coronary angiography for the diagnostic work-up of coronary artery disease: Application of the European Cardiovascular Magnetic Resonance registry data to the German, United Kingdom, Swiss, and United States health care systems. *Journal of Cardiovascular Magnetic Resonance*. 2012;14(1):35.

2. Roos JB, Doshi SN, Konorza T, et al. The cost-effectiveness of a new percutaneous ventricular assist device for high-risk PCI patients: mid-stage evaluation from the European perspective. *Journal of medical economics*. 2013;16(3):381-390.

3. Boldt J, Leber AW, Bonaventura K, et al. Cost-effectiveness of cardiovascular magnetic resonance and single-photon emission computed tomography for diagnosis of coronary artery disease in Germany. *Journal of cardiovascular magnetic resonance*. 2013;15(1):30.

4. Assanelli D, Levaggi R, Carré F, et al. Cost-effectiveness of pre-participation screening of athletes with ECG in Europe and Algeria. *Internal and emergency medicine*. 2015;10(2):143-150.

5. Taylor MJ, Scuffham PA, McCollam PL, Newby DE. Acute coronary syndromes in Europe: 1-year costs and outcomes. *Current medical research and opinion*. 2007;23(3):495-503.

6. *World Health Organization. Health  service delivery costs*. 2011. <http://www.who.int/choice/cost-effectiveness/inputs/health_service/en/>

7. Icks A, Haastert B, Gandjour A, et al. Costs of dialysis—a regional population-based analysis. *Nephrology Dialysis Transplantation*. 2009;25(5):1647-1652.
